# Implementation of Step 7 of the Baby-Friendly Hospital Initiative (BFHI) in Finland: Rooming-in according to mothers and maternity-ward staff

**DOI:** 10.18332/ejm/93771

**Published:** 2018-08-23

**Authors:** Mervi Hakala, Pirjo Kaakinen, Maria Kääriäinen, Risto Bloigu, Leena Hannula, Satu Elo

**Affiliations:** 1 Northern Ostrobothnia Hospital District, Oulaskangas Hospital, Oulainen, Finland; 2 Research Unit of Nursing Science and Health Management, University of Oulu, Oulu, Finland; 3 Medical Research Center (MRC), Oulu University Hospital and University of Oulu, Oulu, Finland; 4 Medical Informatics and Statistics Research Group, University of Oulu, Oulu, Finland; 5 Metropolia University of Applied Sciences, Helsinki, Finland

**Keywords:** breastfeeding, postpartum, quantitative research, roomingin, Baby-Friendly Hospital Initiative, Step 7

## Abstract

**INTRODUCTION:**

Rooming-in is an evidence-based practice during which postpartum mothers and infants stay together. Rooming-in benefits both the mother and infant, and is especially important for breastfeeding. This study aims to describe rooming-in (Step 7 of the BFHI), according to mothers and maternity-ward staff in Finnish maternity hospitals, as well as the factors associated with its implementation.

**METHODS:**

The presented research adopted a cross-sectional study approach. Questionnaires were used to collect data from mothers (n=111) who had given birth and the attending maternity-ward staff (*f*=1554 reported events) at 8 Finnish maternity hospitals. The data were analysed using descriptive statistics, as well as chi-squared, t-test, and Fisher, Mann-Whitney, Kruskal-Wallis tests. Answers to the open-ended questions were analysed using content specifications.

**RESULTS:**

Rooming-in was utilised to a satisfactory extent, especially after vaginal birth. Most of the mothers regarded it as a very positive experience. Rooming-in was delayed mainly because of a mother’s tiredness and the infant’s condition. Factors such as a staff member’s age, work experience, and completion of breastfeeding counselling training (WHO 20-h), a mother’s parity, need for supplementation, and mode of childbirth, were found to be associated with the decision to implement rooming-in.

**CONCLUSIONS:**

Rooming-in should be used more with infants born by caesarean section and primiparous mothers. The need for supplementation clearly increased when roomingin was not employed. The presented information could be crucial for effectively allocating maternity ward resources and demonstrating the importance of rooming-in to a diverse audience of health care professionals.

## INTRODUCTION

The Baby-Friendly Hospital Initiative (BFHI) is meant to support, protect, and promote breastfeeding in facilities providing maternity and infant services^1^. This initiative is a global programme that was launched in 1991 by the World Health Organization (WHO) and UNICEF as a response to an alarming decrease in breastfeeding, and was last revised April 2018. The programme aims to provide new mothers with high-quality clinical care, as well as increase the proportion of mothers who exclusively breastfeed. A practical guideline, termed the ‘Ten Steps to Successful Breastfeeding’, exists for the successful implementation of BFHI in maternity wards^1^.

Numerous studies have shown that compliance to the BFHI programme is related to positive outcomes, including breastfeeding^2-4^. Although mothers now spend less time at the hospital than before, the BFHI includes a period that is crucial for continued, successful breastfeeding. During their time at the hospital, mothers receive the support they will need to continue breastfeeding at home. The counselling and support that health care professionals provide during the first days is pivotal to ensuring that mothers will continue to exclusively breastfeed at home^3,5^. Baby-Friendly certified hospitals provide maternity ward staff with clear instructions on how they can support breastfeeding. As a result, the maternity-ward staff at these hospitals may work differently than maternity-ward staff in non-Baby-Friendly certified hospitals^1^. In this article, a non-Baby-Friendly certified hospital refers to any hospital that does not have a Baby-Friendly hospital certificate. Global statistics show that in 2017 only 10% of infants were born in Baby-Friendly certified hospitals. Only a few hospitals (16%) in Finland are Baby-Friendly certified^6^, but the BFHI Steps are used as general guidelines in every maternity hospital^7,8^.

The proportion of Finnish mothers who exclusively breastfeed has decreased to alarmingly low levels, with only 1% exclusively breastfeeding at six months in 2012 (National Institute for Health and Welfare)^9^. This falls short of the global average, which now stands at 43% and has been increasing^10^, and lower than the averages of other Nordic countries (Sweden 14%, Norway 7%, Denmark 17%, Iceland 13%)^6^. Thus, it is important to understand the current rooming-in situation in Finland, as this knowledge is crucial to promoting exclusive breastfeeding. Rooming-in is an evidence-based practice that is used in hospitals to take care of postpartum mothers and their infants during the postpartum period^11^. Rooming-in means that the postpartum mother and her infant are together in the same room after birth for 24 hours a day^12,13^ and they are cared for as a ‘couple’^14^. Roomingin is divided into full rooming-in (24 h), which means that the infant stays in the mother’s room day and night, and partial rooming-in, during which the infant is in its mother’s room during the day but transferred to the nursery at night^15^. The practice of rooming-in is also Step 7 in the BFHI’s ‘Ten Steps to Successful Breastfeeding’ guideline, which recommends: ‘Enable mothers and their infants to remain together and to practise rooming-in throughout the day and night’^1^. In this study, rooming-in refers to full rooming-in. Rooming-in is especially important for breastfeeding. It has been shown to improve breastfeeding in general^16^, exclusive breastfeeding at the hospital^17^, following discharge^15,18^ and exclusive breastfeeding at 6 months^19^, as well as the duration of breastfeeding^13.^ Rooming-in makes breastfeeding easier for mothers^20^ as it allows frequent day and night feedings^16,21^. Furthermore, infants who stay in the nursery may be more likely to get fed with formula milk than rooming-in infants^22^. Mother-infant proximity and interactions during early postpartum period are important for breastfeeding success and milk production^23^ and these interactions demand mother and infant stay together. Mothers who room-in have shown a positive attitude towards breastfeeding^24^, and were generally satisfied with the rooming-in experience^14^.

Rooming-in is also important for developing a mother’s ability to respond to her infant’s needs^21^ and it facilitates a good start to mother-infant interaction^25-27^. It helps mothers learn and recognise their infant’s hunger cues^28,29^, and mothers gain self-confidence when they can recognise that their infant is comfortable and contented^21,30^. It also facilitates early bonding and helps mother and infant ‘get to know’ each other sooner^20,31^. In addition, rooming-in allows a mother to practice both caring for her infant and breastfeeding in a safe environment^32^. The staff can support and counsel parents at all times^14^, and ensure that parents grasp the importance of keeping their infant close^28,33^. There is evidence that a mother’s oxytocin^34^ and beta-endorphin levels^24^ as well as emotional and physical wellbeing increase during rooming-in^35^, and that it helps women embrace their new role of being a mother^29,36^. Furthermore, it was shown that mothers who room-in are more sensitive towards their infant and respond to its needs lovingly and tenderly^13,20^. Mothers and infants who room-in sleep better and experience less anxiety^37,38^. Moreover, skin-to-skin contact, which is easy to use in rooming-in, has been shown to reduce postpartum depressive feelings and maternal physiological stress^29^. Rooming-in increases closeness and bonding between parents and infant^17,29^, strengthens a mother’s positive attachment to her infant^26^, reduces depressive feelings and maternal physiological stress^29^ and provides the infant with emotional security^33,36^.

Rooming-in reduces the risk of neonatal complications^39^ such as a diabetic mother’s infant hypoglycaemia, morbidity^39^, hyperbilirubinemia^16^ and neonatal abstinence syndrome (NAS), as well as the rate of infant admission to the neonatal intensive care^12,40-42^ and the need for pharmacotherapy^41-43^. Infants cry less^44^ and sleep better^22^ when they are close to their mother. Any separation of a mother and infant disrupts brain development, which is required for bonding. Furthermore, stress hormone levels increase when the infant is away from its mother, possibly destabilising the infant. Preterm infants gain more weight per day when rooming-in^45^, and infants, who have NAS, have shorter stays in the Neonatal Intensive Care Unit (NICU)^12,40,41^, which makes this approach costeffective^14,43^. Rooming-in also benefits nurses, as they are entrusted with higher levels of accountability and can build their competence, which will translate into improvements in staffing flexibility and job satisfaction^14^.

Nevertheless, the negative sides of rooming-in must also be considered. Previous studies have shown that roomingin can interfere with a mother’s need to sleep^38,46^. Mothers who room-in may suffer from fatigue, sleep deprivation and exhaustion, and this is common when a mother is responsible for caring for an unsettled infant during the night^47^. Among mothers who room-in, those who gave birth through a caesarean section reported more postpartum fatigue than mothers who gave birth vaginally. Mothers who experience postpartum fatigue have difficulty performing infant-care activities, which can weaken mother-infant attachment^38,48^. According to Hunter et al.^38^, mothers who cannot determine why their infant is crying or who are unfamiliar with babysoothing skills may experience difficulties in infant care. Rooming-in also presents some risks for infants, as 58% of infant falls happen between midnight and 7 a.m., and 55% of falls occur after a family member falls asleep in a bed or chair^17,35^.

This study aims to describe rooming-in (Step 7 of the BFHI) according to mothers and maternity-ward staff in Finnish maternity hospitals and to identify factors associated with the implementation of rooming-in. The study questions were: ‘What are postpartum mothers’ and maternity ward staff’s perceptions of rooming-in?’ and ‘What background factors are associated with the implementation and duration of rooming-in?’.

## METHODS

### Participants and data collection

Study participants comprised new mothers with their infants and the maternity-ward staff (midwives, childminders/ practical nurses, nurses and students) who cared for the mothers in maternity wards and NICUs in 8 hospitals in Finland. The hospitals were chosen by stratified random sampling, so that the study included two representative hospitals for each of the following types of hospitals defined by the National Institute for Health and Welfare: under 750 deliveries; 750–1500 deliveries; over 1500 deliveries; and university hospital^49^. Two of the eight hospitals had a BFHI certificate. Each maternity hospital had a designated contact person who shared information about the study.

**Figure 1:**
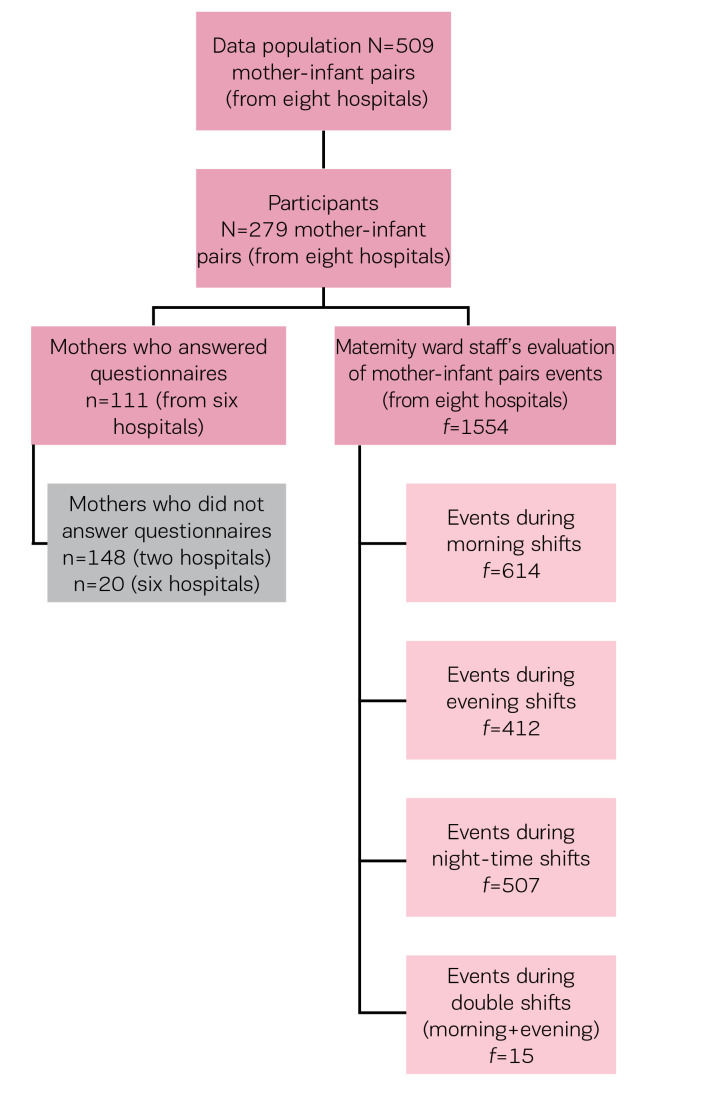
Research frame

This research employed a cross-sectional study design. Data were collected using two different questionnaires (one for new mothers and the other for maternity-ward staff) during one week in Spring 2014. The research frame is presented in Figure 1. The study population comprised mothers (n=509) who had given birth during the data collection period and stayed in maternity wards or NICUs, as well as the maternityward staff who attended to these new mothers. Of the new mothers, 279 decided to participate in the study. However, two of the studied hospitals did not allow new mothers to answer the questionnaire. As a result, only 111 new mothers from 6 hospitals answered the questionnaire, representing a response rate of 59%. The mothers filled in the questionnaire before discharge. An unknown number of maternity-ward staff filled in a total of 1554 questionnaires concerning the 279 mother-infant pairs during the data collection period. The results concerning the maternity-ward staff questionnaire will be presented using the abbreviation f. This is because it is possible the same midwife or nurse filled in the questionnaire multiple times. For each mother-infant pair (n=279) included in the study, the maternity-ward staff member who took care of them filled in a questionnaire after every work shift, which means that three questionnaires per mother-infant pair were filled in each day.

### Questionnaires

The questionnaire for mothers as well as the separate questionnaire for maternity-ward staff were developed specifically for this study. The questionnaire for mothers included 31 questions, with 7 background questions and 3 questions about rooming-in. The questionnaire for maternityward staff included 27 questions, of which 9 were background questions and 4 specifically concerned rooming-in. Other items covered different fields of BFHI, such as skin-to-skin contact, initial breastfeeding and exclusive breastfeeding. The results for BFHI Steps other than rooming-in (Step 7) are reported elsewhere^7,8^. Mothers and maternity-ward staff responded to questions that were multiple choice, graded on a five- or six-point Likert scale, dichotomous (yes/no) or open-ended (Table 1). Data collected from midwives working in the delivery room were used to describe the details of births (Table 2).

**Table 1:** Content and type of questions in mothers’ and maternity ward staffs’ questionnaires

*Type of question*	*Mothers’ research topics*	*Maternity ward staff’s research topics*
**Open-ended**	age, parity, infant’s birthweight, barriers to full rooming-in, success of breastfeeding, amount of supplementation during hospitalization, reasons that blood tests were taken from infants, hospitalization days	hospital (name, size of hospital, Baby-Friendly hospital or non-Baby-Friendly hospital), age, years of work experience, staff’s responsibility during shift, reasons for using skin-to-skin contact, barriers to full rooming-in, amount of supplementation, times of blood test, bed-day, reasons for supplementation, reasons for taking blood test (infant)
**Multiple-choice**	mode of childbirth, pain relief, starting time of initial breastfeeding, size of room at ward, quality of supplementation	occupation, work shift, duration that full rooming-in was not used, quality of supplementation, method of supplementation
**Likert scale (five-point)**	experience of childbirth, success of initial breastfeeding	
**Likert scale (six-point)**	experience of skin-to-skin contact, experience of initial breastfeeding, experience of roomingin, experience of breastfeeding, experience of supplementation	
**Dichotomous**	implementation of skin-to-skin contact, need for initial breastfeeding counselling, adequacy of initial breastfeeding counselling, continuity of initial breastfeeding, implementation of full roomingin, need for nipple shield, need for pacifier, need for supplementation, need for blood test (infant), adequacy of breastfeeding counselling	completion of WHO 20-h breastfeeding counselling training, enough knowledge of breastfeeding counselling, opinion of full rooming-in, implementation of full rooming-in, adequate time to provide breastfeeding counselling, success of breastfeeding, need for breastfeeding counselling, need for nipple shield, need for supplementation, need for blood test (infant), implementation of skin-to-skin contact

**Table 2:** Background information for childbirths (N=279) according to midwives in labour room

*Background*	*n*	*%*	*range*
**Mothers’ parity**			1–16
I-parturient	125	46	
II-parturient	96	35.3	
III-parturient	28	10.3	
IV-parturient	12	4.4	
V-parturient or more	11	4	
**Mode of childbirth**			
normal/assisted vaginal	232	86	
caesarean section	38	14	
**Pregnancy weeks at the time of birth**			30–42
36 or less	14	5.2	
37–38	55	20.3	
39–40	143	52.7	
41–42	59	21.8	

The questionnaires included 12 questions from questionnaires that had been used earlier^50,51^, as well as questions created by expert panels. The content validity of the questionnaires was evaluated by three different expert panels: committee members from the Federation of Finnish Midwives (n=7) (content experts, superiors, midwives); midwives (n=6); and experts in instrument development for nursing sciences (n=3) (professor, university lecturer, consultant). A few changes were made to the questionnaires after the evaluation. After an evaluation by the expert panels, the pilot questionnaire was distributed in three maternity wards. Based on the results, some changes were made, for example, two questions were taken out and some questions were clarified in the maternity-ward staff questionnaire. Four of the questions in the questionnaire for mothers were clarified following the pilot experiment. The questionnaires were formulated in both Finnish and Swedish, which are official languages in Finland. According to the evaluations, the questionnaire for maternity-ward staff was easy to fill in, while performing other hospital duties.

### Ethical issues

Permission to perform the study was requested and received from all the hospitals that participated in the study. Approval was also sought from the Regional Ethics Committee of the Northern Ostrobothnia Hospital District and Turku Clinical Research Centre, yet both unambiguously reported that the study does not require the approval of the Research Ethics Committees based on the Medical Research Act (1999/488). They stated that it was sufficient to receive permission from each participating hospital^52^. One hospital district did not give permission for the mothers to be included in the study but allowed maternity-ward staff to participate.

The low response rate of postpartum mothers could be explained by a mother’s condition after giving birth and the strong desire to concentrate on caring for her infant. Participation in the study was voluntary. Maternity-ward staff were informed about the study by a contact person both orally and via a cover letter. Mothers were informed about the study by midwives both orally and via a cover letter. All of the participating maternity-ward staff and mothers gave their informed oral consent based on the provided information. The main researcher did not establish a person register, and the study ensured the anonymity of every participant^53^. The questionnaires and the data they produced were coded in such a way that the researchers could handle the data without losing information about which mother-infant pair was described.

### Data analysis

The data were analysed using SPSS Statistics for Windows (version 24.0, IBM, Armonk, NY)^54^. The data were first examined using descriptive statistics (frequencies, percentages). Differences between background variables and main (rooming-in) variables were tested using chi-squared, t-test, and Fisher, Mann-Whitney, Kruskal-Wallis tests. All of the results presented in this study are statistically significant (p<0.05)^53^. Open-ended questions were analysed based on content specifications^55,56^. Answers to open questions were typically short, consisting of only a couple words or short paragraphs. Therefore, it was not possible to perform a deep content analysis. Content specification was adopted as qualitative answers describe phenomena better than quantitative answers^57^. Content was organized into two categories — category and subcategory. Participants with missing data were excluded. Results regarding new mothers are described using the number of observations (n), while nursing events are described using the amount of reported effects (f) in the questionnaires filled in by maternity-ward staff after each work shift. Results of open-ended questions are presented using q, which describes the number of times a certain word, phrase or concept appeared in the open-ended questions.

## RESULTS

### Characteristics of participants

The mean age of mothers (n=111) was 30 years (range 19– 46 years). Of the mothers that answered the questionnaire, 82% (n=91) gave normal birth vaginally, 9% (n=10) vacuumassisted vaginally, 1.8% (n=2) breech birth vaginally and 7.2% (n=8) by caesarean section. About a third (31%, n=34) of the mothers were primiparous. One set of twins was included in the data. Mothers stayed at the maternity-ward in either single rooms (n=13, 12%), double rooms (n=46, 42%), a family room (n=5, 4%), or rooms with three, four or six beds (n=46, 42%). The average hospital stay lasted 2.6 days (range 1–8 days).

The characteristics of the maternity-ward staff are presented in Table 3. The average staff member participating in this study was 43 years old and had 16 years of work experience. Most of the questionnaires were filled in by midwifes (n=961, 67%), and almost all of the respondents (97%) had completed breastfeeding counselling training (WHO 20-h). The maternity-ward staff were responsible for an average of 4.5 infants per shift (range 0–29). According to the maternity-ward staff, the average length of a mother’s pregnancy was 39 weeks (range 30–42 weeks), with the average infant weight at 3500 g (range 1890–4660 g). Furthermore, the maternity-ward staff reported that 86 % (n=232) of the mothers included in the study gave birth vaginally and 46 % (n=125) of them were primiparous (Table 2). These results can be compared with the responses of mothers presented above.

**Table 3:** Background information for maternity ward staff (f=1554)

*Background*	*n*	*%*	*range*
**Occupation of staff**			
midwives	961	66.7	
children’s practical nurses and practical nurses	404	28	
nurses	76	5	
students	5	0.3	
**Age of maternity ward staff, years**			20–64
20–30	320	21	
31–40	349	23	
41–50	318	20	
51–60	499	32	
over 60	62	4	
**Years of work experience**			0–37
0–10	638	43	
11–20	272	18	
21–30	380	25	
31–40	209	14	
**Shift**			
morning	614	40	
evening	412	26	
night	507	33	
double (morning + evening)	15	1	
**Staff with WHO 20-h breastfeeding counselling training**			
Yes	1502	97	
No	52	3	
**Staff’s responsibility of infants during shift**			
0–5 infants/nurse	1132	74	
6–10 infants/nurse	348	23	
11 or over infants/nurse	50	3	

### Mothers’ evaluation of rooming-in and factors related to its implementation

Most mothers (86%, n=94) agreed that rooming-in was used to a suitable extent. The size of the room (p=0.046) was associated with whether or not rooming-in was employed. Rooming-in was always used when the mother stayed in the family room, and in 93% of cases in which the mother stayed in a room with three to six beds. However, only 74% of mothers in double rooms roomed-in with their infants. The infants who roomed-in needed less supplementation than the infants in the nursery (p=0.025).

In all, 81% of mothers experienced rooming-in as positive and most multiparous (p=0.013) mothers reported it to be a highly positive experience. A positive perception of skinto-skin contact after labour was associated (p=0.001) with a positive experience of rooming-in.

### Maternity-ward staff’s evaluation of rooming-in and factors associated with its implementation

The maternity-ward staff (*f*=1447) reported that rooming-in was used following 91% of births. The staff member’s age (p=0.049), work experience (p=0.022), and completion of breastfeeding counselling training (WHO 20-h) (p=0.001) were associated with a mother’s decision to room-in with her infant (Table 4). Staff members who were over 60 years old and had extensive work experience implemented rooming-in more often than their younger, less experienced counterparts. Maternity-ward staff who had worked for over 30 years suggested that mother-infant pairs should room-in more often than staff with 0–10 years of experience. Furthermore, maternity-ward staff who had completed breastfeeding counselling training (WHO 20-h) were more inclined to apply the rooming-in approach than staff who had not completed this training. The implementation of rooming-in was also associated with staff having adequate time to provide breastfeeding counselling (p=0.000) and the mode of childbirth (p=0.000) (Table 4). When the staff had adequate time to implement breastfeeding counselling, rooming-in was employed in 93% of the cases. Rooming-in was more commonly used after a vaginal birth than after a caesarean section. Rooming-in was more positively perceived when it was used after a vaginal delivery than after a caesarean section (p=0.026). According to maternity ward-staff, there was a strong association between implementation of rooming-in and skin-to-skin contact at the postpartum ward (p=0.014) (Table 4).

**Table 4:** Relationship between maternity ward staff background variables and the implementation, duration and professional opinion of rooming-in

	*Implementation of rooming-in*	*Duration of rooming-in*	*Opinion of full rooming-in*
**Age**	p=0.049	ns*	ns*
**Occupation**	ns*	ns*	p=0.004
**Years working as a nurse**	p=0.022	ns*	ns*
**Work shift**	ns*	p=0.002	ns*
**Breastfeeding counselling training (WHO 20 h)**	p=0.001	ns*	ns*
**Implementation of skin-to-skin contact**	p=0.014	ns*	ns*
**Implementation of rooming-in**	x	p=0.000	p=0.021
**Adequate time to provide breastfeeding counselling**	p=0.000	p=0.006	ns*
**Size of hospital**	ns*	p=0.006	ns*
**Mother’s parity**	ns*	p=0.027	ns*
**Baby Friendly certification**	ns*	p=0.005	ns*
**Mode of childbirth**	p=0.000	p=0.009	p=0.026

* ns= p>0.05

A staff member’s occupation (p=0.004) was associated with their opinion of rooming-in (Table 4). Midwives and nursing students were more satisfied with rooming-in than children’s-practical-nurses/practical-nurses and nurses. The decision to implement rooming-in (p=0.021) and mode of childbirth (p=0.026) were also associated with a staff member’s opinion of full rooming-in (Table 4).

### The duration of rooming-in along with relevant factors

The maternity-ward staff took care of the infants for an average of 2.3 hours per shift. Most of the infants were with maternity-ward staff for either under one hour (36%, *f*=64) or for over five hours (26%, *f*=45). Work shift (p=0.002), work load (p=0.006) and the mode of child birth (p=0.009) were all associated with the duration of rooming-in (Table 4). Nurses working double or night shifts reported longer durations during which infants were away from their mothers than nurses working other shifts. Infants were away from their mothers for over five hours in 50% and 35% of cases when maternity-ward staff were working double and night shifts, respectively. The infant was with its mother for a longer period of time when maternity-ward staff members had enough time during their shift to provide breastfeeding counselling than when either only partial counselling was implemented, or the infant was born by caesarean section.

Infants at university hospitals were more often out of the room for over five hours than infants at hospitals with 750–1500 deliveries, and this difference was statistically significant (p=0.006). Furthermore, infants at Baby-Friendly certified hospitals were more often out of the room for over one hour than infants at non-Baby-Friendly certified hospitals (p=0.005). Primiparous mothers’ infants were out of the room for three hours more often than the infants of multiparous mothers (p=0.027). The duration of rooming-in was found to be associated with implementation of roomingin (p=0.000) (Table 4).

### Barriers to rooming-in according to mothers and maternity-ward staff

Barriers to rooming-in were identified from answers to open-ended questions, and both mothers (n=17) and maternity-ward staff (*f*=137) described multiple reasons for why rooming-in was unsuccessful. Most of the barriers (q=73) were related to a mother’s postpartum condition, namely, fatigue, need to rest and sleep (q=24), basic needs (q=25) (e.g. shower) and consequences of caesarean section (q=11) (e.g. pain), with the two most common barriers being a mother’s fatigue, need to rest and sleep, and infant admittance to the NICU (Table 5). All of the reported barriers to rooming-in are detailed in Table 5.

**Table 5:** Barriers to rooming-in according to mothers and maternity ward staff

*Mothers*	*q*	*%*	*Maternity ward staff*	*q*	*%*
Mother’s need to rest and sleep, fatigue	8	40	Mother’s need to rest and sleep, fatigue	31	18.7
Infant care in NICU	6	30	Infant care in NICU	33	19.9
Caesarean section	2	10	Caesarean section	11	6.6
Restless infant	2	10	Restless infant	11	6.6
Insufficiency of breast milk	1	5	Infant spends a long time at breast	2	1.2
Infant’s light therapy	1	5	Infant’s light therapy	11	6.6
			Mother’s basic needs	25	15
			Mother’s problems	22	13.2
			Mother out of the ward	10	6
			Infant’s treatment	10	6

## DISCUSSION

When the results from the mothers and maternity-ward staff included in this study are considered together, it can be stated that rooming-in was implemented to a satisfactory extent, which is comparable to an earlier study concerning Southern Finland^30^.

Early skin-to-skin contact and initial breastfeeding after labour were found to be associated with the implementation of rooming-in. This practice enables positive bonding between the infant and mother^28^ and, together with rooming-in, promotes mother-infant interaction^58^ along with sustained, long-term exclusive breastfeeding^3,18^. This study also identified an association between early skin-toskin contact following labour and a positive experience of rooming-in. An infant gets the best start to its life through the many benefits of rooming-in^17^, and all maternity-ward staff should understand and apply this concept in their work. One study has suggested that non-separation benefits fullterm infants and their mothers^28^, as well as maternity-ward staff^14^. The maternity-ward staff unanimously felt that full rooming-in is good hospital practice. One study has found that full rooming-in increases nurses’ job satisfaction and affords them more responsibility^14^. A staff member’s occupation was associated with their opinion of roomingin, i.e. all midwives felt that it is good practice but staff representing other occupations did not share unanimously a positive perception. This may be because the education of these other professions does not concentrate on the benefits of breastfeeding and keeping the mother and infant together, as much as the curriculum of midwives. An evidence-based review by Jaafar et al.^18^ found limited support for the claim that rooming-in is better than separation after birth in terms of exclusive breastfeeding once the mother is home. The outcomes showed that rooming-in translated to a higher frequency of breastfeeding and improved rates of exclusive breastfeeding four days postpartum. Nevertheless, the research included a brief study period and acknowledged that further information about the realised benefits of rooming-in are need, which the presented study doesn’t provide.

The age and work experience of maternity-ward staff were both associated with the decision to implement roomingin. Extensive work experience underlies taking good care of mother-infant couples. Breastfeeding counselling training (20-h) seems to be a good form of education for maternityward staff as it was found to be associated with how often rooming-in is utilised. However, this education should be mandatory for all maternity-ward staff rather than just the midwives who attend to mother-infant couples, as roomingin rates were higher when staff members reported having enough time to provide breastfeeding counselling. Insufficient resources for breastfeeding counselling require wards to be well organised and to concentrate on mothers who are at risk of not breastfeeding their infant. The mode of childbirth also affected whether or not rooming-in was implemented. In this study, rooming-in was very common after normal vaginal childbirth, and most health care professionals felt that it is more appropriate after vaginal delivery than following a caesarean section. However, the separation of mother and infant after a caesarean section increases stress hormone levels in infants, and these infants also have lower betaendorphin levels than infants born through vaginal deliveries^32^. Following early separation, a mother’s oxytocin and prolactin levels decrease while her epinephrine levels increase, which can lead to increased stress^32^. Mothers who deliver through a caesarean section need more help and support during rooming-in. Maternity-ward staff are competent in providing breastfeeding assistance to mothers who had a caesarean section, especially if they have completed breastfeeding counselling training (WHO 20-h), yet it is sometimes difficult to realise that a mother needs assistance^24^.

When full rooming-in was not used, the infant was out of the mother’s room for an average of two hours per shift. Most of the breaks lasted less than one hour and were the result of a mother’s basic needs. Infants in the nursery are more likely to receive supplementation than infants who room-in^22^, and this is not beneficial for the frequency of breastfeeding. The results presented in this study agree with previous findings. The most dangerous consequence of an infant receiving supplementation at the hospital is that the mother may feel that she does not have enough breast milk to feed her infant. Infants in the nursery also used a pacifier more than infants who roomed-in, but this finding was not statistically significant. The finding demonstrates that infants’ sucking needs are sated by a pacifier. Long-term exclusive breastfeeding requires a large amount of emotional support, self-efficacy and good experiences from the hospital so that mothers continue breastfeeding at home^30^. Roomingin can be essential to a mother learning her infant’s hunger cues^28^ and being able to immediately respond to them^21^; it also facilitates frequent feeding^16,18^ and helps mothers to ‘get to know’ their infants sooner^31^. As a result, rooming-in increases exclusive breastfeeding^16^, makes breastfeeding easier^20^ and builds a loving and tender relationship between infant and mother^28,29,41^.

The BFHI programme aims to support, promote and protect exclusive breastfeeding in every hospital around the world^59^. One surprising result in this study was that BFHIcertified hospitals did not stand out in terms of rooming-in practice. Infants in BFHI-certified hospitals were out of their rooms more than infants in non-BFHI-certified hospitals. This demonstrates that all Finnish hospitals have a similar stance regarding Step 7 of BFHI and that our birth and postpartum culture is well-aligned with the BFHI advice. In Finland all hospitals work with postpartum mothers and their infants in a quite similar way, although some of them are BFHI-certified. The culture of interactions during postpartum time are deep rooted in a country. We did, however, find that hospital size affects rooming-in utilisation in Finland, as rooming-in was used significantly more at hospitals with 750–1500 childbirths than at university hospitals. This finding may be explained by smaller hospitals having more single, family and double rooms, which would increase connectivity and thus rooming-in. The size of the room also influenced the implementation of rooming-in, as rooming-in was more common in double rooms than in smaller (single) or bigger (three to six beds) rooms. The lower rates of rooming-in in the largest rooms may have been a result of mothers feeling pressure to allow the other mothers an opportunity to sleep. One important factor to increase rooming-in may be family support for it. Nowadays fathers spend more time at wards and may care infants with mothers and so increase roomingin. Our study did not research this factor.

The results clearly demonstrated that a mother’s need to sleep and rest is a main barrier to successful rooming-in, and this problem has been highlighted by another study^46^. Mothers who were primiparous, gave birth by caesarean section^38,48^ and/or had a complicated and prolonged labour were more at risk to postpartum fatigue. Any mother who has undergone a difficult labour or caesarean section requires ample rest so that they can fully recover both physically and psychologically^21^. Hospitals are responsible for ensuring a mother’s wellbeing, and partial rooming-in may be a solution for mothers experiencing postpartum fatigue. It is good practice to allow a mother to leave an infant at the nursery for a short time when she is too tired or stressed, and this has been shown to make exclusive breastfeeding less demanding^15^ and to improve a mother’s self-efficacy^30^. In this study, infants were out of the room for the longest periods at nights and during double shifts. This demonstrates that the studied hospitals are actively working to prevent mothers’ postpartum fatigue. Exclusive breastfeeding is also possible with partial rooming-in. Maternity-ward staff can provide breastfeeding counselling to help mothers understand the importance of exclusively breastfeeding at night and that they can manage even when they are tired. Other studies have presented contradictory results regarding complaints about rooming-in and a mother’s fatigue. Some of these studies found that mothers who practice rooming-in sleep better and have less anxiety^37,38^. For this reason, maternityward staff have to be adept at recognising differences among mother-infant couples. In this way, they can help mothers decide whether rooming-in is the best choice for them and their infant. In this study, maternity-ward staff reported not having sufficient time to provide complete breastfeeding counselling, which is a concern. Under time pressure, the maternity-ward staff concentrated on counselling the essential mothers, i.e. primiparous women and mothers who had delivered via caesarean section. A possible reason for this decision is that if these mothers are supported well following the birth of their first child, then they will not need as much support during future deliveries.

Infants’ treatment in the nursery or NICU prevents the implementation of rooming-in. This could explain why not all of the mothers included in this study roomed-in with their infants. Based on evidence from previous research, this needs to change in the future. Numerous studies have shown that it is beneficial for mother and infant to be close. For this reason, hospitals should seriously consider adding family rooms to the NICU. The possibility of rooming-in in the NICU could, according to previous literature, have beneficial effects in terms of NAS treatment^12,41^, prevalence of neonatal hypoglycaemia^39^ and infections^60^, as well as length of NICU stay^41^. Postnatal NAS can occur following the discontinuation of drug use by the infant’s mother and is a typical problem in infants born to mothers who are dependent on opioids^41^. Many other reasons prevented rooming-in but they occurred less often.

### Limitations

This study focused only on one country and included a limited number of participants. The maternity-ward staff’s questionnaires could have been filled in by the same personnel multiple times, therefore having the same results reported, which may influence some of the parameters evaluated in the study. Hence further research is needed with a more targeted population to obtain concrete information on the impact of rooming in Finnish maternity wards.

## CONCLUSIONS

This study has shown that maternity wards in Finnish hospitals implement the practice of rooming-in to a satisfactory extent, as a substantial number of mother-infant couples are together during the postpartum period. This allows mothers and infants to get the best possible start to breastfeeding. Moreover, infants in the nursery received more formula milk than infants who roomed-in. This study supports increased implementation of the BFHI programme across Finnish hospitals, and contributes knowledge pertaining to Step 7 (rooming-in) of the initiative. Roomingin was used to a large extent with multiparous mothers and after vaginal birth. One clear challenge is how maternity-ward staff could increase rooming-in among primiparous women and mothers who delivered via caesarean section. More research is necessary before solutions to this challenge can be postulated. The current situation in Finland is promising, but there are still some problems that need to be addressed.

Our main recommendation would be that all maternity-ward staff clearly understand the importance of and their role in implementing rooming-in with the end goal being long-term, exclusive breastfeeding. A key part of this is that all staff members have completed the WHO 20-h breastfeeding counselling training, as staff who have completed this education implement rooming-in more often than staff who lack this training.
